# Halophytes As Bioenergy Crops

**DOI:** 10.3389/fpls.2016.01372

**Published:** 2016-09-13

**Authors:** Rita Sharma, Silas Wungrampha, Vinay Singh, Ashwani Pareek, Manoj K. Sharma

**Affiliations:** ^1^School of Computational and Integrative Sciences, Jawaharlal Nehru UniversityNew Delhi, India; ^2^Stress Physiology and Molecular Biology Laboratory, School of Life Sciences, Jawaharlal Nehru UniversityNew Delhi, India; ^3^School of Biotechnology, Jawaharlal Nehru UniversityNew Delhi, India

**Keywords:** biofuel, halophytes, lignocellulosic biomass, oilseed, salinity

## Abstract

Shrinking arable land due to soil salinization and, depleting fresh water resources pose serious worldwide constraints to crop productivity. A vision of using plant feedstock for biofuel production can only be realized if we can identify alternate species that can be grown on saline soils and therefore, would not compete for the resources required for conventional agriculture. Halophytes have remarkable ability to grow under high salinity conditions. They can be irrigated with seawater without compromising their biomass and seed yields making them good alternate candidates as bioenergy crops. Both oil produced from the seeds and the lignocellulosic biomass of halophytes can be utilized for biofuel production. Several researchers across the globe have recognized this potential and assessed several halophytes for their tolerance to salt, seed oil contents and composition of their lignocellulosic biomass. Here, we review current advances and highlight the key species of halophytes analyzed for this purpose. We have critically assessed the challenges and opportunities associated with using halophytes as bioenergy crops.

## Introduction

Uninterrupted supply of energy is a prerequisite to global technological and economic development. However, with increasing population and energy consumption rates, fossil fuel-based energy resources may only last a few more decades. Also, limited stocks and environmental hazards associated with the usage of fossil fuels have led to global interest in exploring renewable and greener sources of energy (van der Weijde et al., 2013). Plant-based biofuels are greener alternative to fossil fuels with potential to mitigate global warming and climate change (Sagar and Kartha, 2007).

Depending upon the source of the raw material, biofuels are classified into different generations. The first generation biofuels are derived from plant sugars, starchy grains and vegetable oils using conventional technologies (Naik et al., 2010). However, since these plant products are also used as animal feed or food, there is a growing concern that their diversion to produce biofuels may lead to global food shortages and, rise in food prices. To deal with the food vs. fuel dilemma, interest was shifted to use of non-food biomass for the production of second generation biofuels (Chundawat et al., 2011). The source of second-generation biofuels could be lignocellulosic biomass or non-food oilseed crops, for example *Pongamia*, *Jatropha*, etc. The lignocellulosic biomass includes agricultural waste in the form of crop straw, husks, forest residue or dedicated perennial grasses such as switchgrass and *Miscanthus*, grown on marginal lands (Hadar, 2013). The production of fuel from these sources has huge potential to minimize environmental footprint, improve soil quality and create high-skilled jobs in the area of technology development and processing. However, high lignin content in these crops is a major cause of recalcitrance for conversion to ethanol. Also, the technology for biochemical conversion of lignocellulosic biomass is expensive and still under development (Simmons et al., 2008; Chundawat et al., 2011). Major limitation with these crops is that they would create an unwarranted and undesirable competition for arable land and fresh water resources used for conventional agriculture. Therefore, large scale planting of bioenergy crops, requiring fresh water resources, may not be feasible (Foley et al., 2005; Koonin, 2006; Kennedy, 2007).

In spite of the fact that over 70% of the earth is covered with water, majority is not suitable for irrigation or human consumption due to high salinity. Further, we have limited area of arable land and its further salinization due to continuous irrigation (that brings dissolved salts) is a major constraint to agricultural production (Flowers and Colmer, 2008). According to a recent estimate, salinized land has increased from ∼45 to ∼62 million ha in last two decades, which is about 20% of the globally irrigated land (Qadir et al., 2014). Over 800 million hectare of the world comprising coastal tideland is also affected by salinity. Clearly, if we can utilize this huge amount of unused niche space for growing biofuel feedstocks that can be irrigated with seawater, we can actually overcome major hurdles associated with farming for biofuels (Rozema and Flowers, 2008).

Based on his experiments in Israel, the ecologist Hugo Boyko showed that several plant species can be irrigated with the salt water (Boyko, 1967). His idea stimulated a global interest to explore the constraints posed by salinity and led to systematic identification of plant species that can grow under saline conditions (Malcolm, 1969; Nielsen et al., 1977; Gallagher, 1985; Pasternak et al., 1985; Glenn et al., 1996). The unique category of plants that can grow, reproduce and thrive well in habitats with very high salt concentrations where most of the other plant species don’t survive, are widely known as halophytes. Recently, Ventura et al. (2014) have reviewed the history and scope of halophyte-based agriculture. The tolerance of halophytes to salt varies in different species and stages of development. Halophytes are widely distributed in the coastal regions, marshy soils, mangroves swamps, estuaries, and saline semi deserts (O’leary and Glenn, 1994; Glenn et al., 1999). They can flourish well in soils ranging from normal to severely saline. Some halophytes can even grow well at salt concentrations higher than that of seawater (>500 mM; Sabovljevic and Sabovljevic, 2007). Interestingly, dicot halophytes are more tolerant as compared to monocot species and exhibit optimal growth in 100–200 mM NaCl, whereas, monocotyledonous halophytes show optimal growth in 50–100 mM of NaCl (Bell and O’Leary, 2003; Flowers and Colmer, 2008).

The number of halophyte species is estimated to range from 2000 to 3000 (Sabovljevic and Sabovljevic, 2007) and majority of them belong to angiosperms. During 1980s, James Aronson compiled a comprehensive database of 1554 halophyte species, termed as HALOPH (Aronson and Whitehead, 1989). This has recently been converted to an interactive eHALOPH database (Santos et al., 2016) that also provides a bibliography of literature available for the halophytes.

Although, halophytes represent less than 2% of the land plant population, they can be used for several commercial purposes such as vegetables, medicinal value, ornamental landscaping, environmental protection and wild life support (Flowers and Colmer, 2008; Cassaniti and Romano, 2011; Koyro et al., 2011; Ladeiro, 2012; Cassaniti et al., 2013; Panta et al., 2014; Ventura et al., 2014). Halophytes have a huge potential to rehabilitate the salt-affected lands and in phytoremediation of polluted soils (Hasanuzzaman et al., 2014; Panta et al., 2014). Due to their unique ability to thrive in saline soils and positive impact on environment, halophytes are potentially good candidates for biofuel production. Some of the halophytes, known as salt accumulators, store salts in their organs as an adaptive mechanism; while others have developed ways to exclude it (Touchette et al., 2009). The salt excluders are in general better choice for biofuels, because non-combustible content of the biomass produced from accumulators may lead to fouling problems.

Due to their unlimited potential, several halophytes species have been screened for large-scale production in different parts of the world and to breed new crops for saline lands. Xian-zhao et al. (2012) reviewed the distribution of major halophytic energy plants in coastal zone of China and assessed their potential for developing energy plants for bioethanol production. Similar surveys have been done in other countries leading to identification of potential halophyte species. Some of the most promising genera are *Salicornia* (glasswort), *Suaeda* (sea-blite), *Atriplex* (saltbush), arid salt grass *Distichlis* and succulent leaved ground cover *Batis* (Squires and Ayoub, 1994; Glenn et al., 1998; Parida and Das, 2005; Rozema and Flowers, 2008; Abideen et al., 2011). Both oilseeds and lignocellulosic biomass produced by halophytes can be used for the production of biofuels. **Table 1** summarizes the halophytes that have been screened for the oil content and composition of lignocellulosic biomass. In several species like *Salicornia bigelovii*, both seed oil and lignocellulosic biomass can be effectively converted into biofuels (Panta et al., 2014). Here, we have summarized the current status of biofuel research with halophytes and highlighted major challenges and opportunities in the area.

**Table 1:** List of Halophyte species assessed for oil and lignocellulosic biomass yields.

S. No.	Name of the Plant	Family	Quantity (% of total dry weight)	Reference
**(A) Halophytes primarily used for oil extraction from seeds**				
1	*Descurainaia sophia*	Brassicaceae	44.17%	Peng et al., 1997
2	*Sarcobatus vermiculatus*	Sarcobataceae	17.5%	Weber et al., 2001
3	*Suaeda torreyana*	Amaranthaceae	25.25%	Weber et al., 2001
4	*Allenrolfea occidentialis*		14%	Weber et al., 2001
5	*Atriplex heterosperma*		15.8%	Weber et al., 2001
6	*Halogeton glomeratus*		24.7%	Weber et al., 2001
7	*Atriplex rosea L*		12.9%	Weber et al., 2001
8	*Kochia scoparia*		9.7%	Weber et al., 2001
9	*Kosteletzkya virginica*	Malvaceae	30%	He et al., 2003
10	*Alhagi maurorum*	Papilionaceae	21.9%	Abideen et al., 2012
11	*Arthrocnemum macrostachyum*	Amaranthaceae	25%	Weber et al., 2007
12	*Salicornia bigelovii*	Amaranthaceae	30%	Weber et al., 2007
13	*Haloxylon stocksii*	Amaranthaceae	22.7%	Weber et al., 2007
14	*Halopyrum mucronatum*	Poaceae	22.7%	Weber et al., 2007
15	*Cressa cretica*	Convolvulaceae	22.3%	Weber et al., 2007
16	*Suaeda glauca*	Amaranthaceae	25%	Du et al., 2009
17	*Suaeda salsa*	Amaranthaceae	22%	Mo and Li, 2010
18	*Ricinus communis*	Euphorbiaceae	47–55%	Zhou et al., 2010
19	*Crithmum maritimum*	Apiaceae	45%	Atia et al., 2010
20	*Helianthus annuus*	Asteraceae	35–52%	Chen and He, 2011
21	*Suaeda aralocaspica*	Amaranthaceae	30%	Wang et al., 2012
22	*Suaeda fruticosa*	Amaranthaceae	25%	Shahi et al., 2013
23	*Kosteletzkya pentacarpos*	Malvaceae	18–22%	Ventura et al., 2014

**S. No.**	**Name of the Plant**	**Family**	**Cell Wall Composition**	**Reference**

**(B) Halophytes primarily used for lignocellulosic biomass (CL, Cellulose; HC, Hemicellulose and LG, Lignin)**				
1	*Panicum virgatum*	Poaceae	45% CL, 31% HC and 12% LG	McLaughlin and Kszos, 2005
2	*Phragmites australis*	Poaceae	50% CL and 17% LG	Yan et al., 2010
3	*Aeluropus lagopoides*	Poaceae	26.67% CL, 29.33% HC and 7.67% LG	Abideen et al., 2011
4	*Aerva javanica*	Amaranthaceae	15.67% CL, 13.33% HC and 6.33% LG	Abideen et al., 2011
5	*Arthrocnemum indicum*	Amaranthaceae	11.33% CL, 13.00% HC and 7.00% LG	Abideen et al., 2011
6	*Calotropis procera*	Apocynaceae	12.33% CL, 11.00% HC and 5.00% LG	Abideen et al., 2011
7	*Cenchrus ciliaris*	Poaceae	22.67% CL, 23.17% HC and 7.00% LG	Abideen et al., 2011
8	*Chloris barbata*	Poaceae	25.33% CL, 23.00% HC and 8.33% LG	Abideen et al., 2011
9	*Desmostachya bipinnata*	Poaceae	26.67% CL, 24.68% HC and 6.67% LG	Abideen et al., 2011
10	*Dichanthium annulatum*	Poaceae	19.00% CL, 24.33% HC and 7.00% LG	Abideen et al., 2011
11	*Eleusine indica*	Poaceae	22.00% CL, 29.67% HC and 7.00% LG	Abideen et al., 2011
12	*Halopyrum mucronatum*	Poaceae	37.00% CL, 28.67% HC and 5.00% LG	Abideen et al., 2011
13	*Ipomea pes-caprae*	Convolvulaceae	12.67% CL, 17.00% HC and 5.33% LG	Abideen et al., 2011
14	*Lasiurus scindicus*	Poaceae	24.67% CL, 29.67% HC and 6.00% LG	Abideen et al., 2011
15	*Panicum turgidum*	Poaceae	28.00% CL, 27.97% HC and 6.00% LG	Abideen et al., 2011
16	*Paspalum paspaloides*	Poaceae	20.33% CL, 33% HC and 2.33% LG	Abideen et al., 2011
17	*Phragmites karka*	Poaceae	26.00% CL, 29.00% HC and 10.33% LG	Abideen et al., 2011
18	*Salsola imbricata*	Amaranthaceae	9.00% CL, 18.33% HC and 2.67% LG	Abideen et al., 2011
19	*Salvadora persica*	Salvadoraceae	22.00% CL, 13.33% HC and 7.00% LG	Abideen et al., 2011
20	*Sporobolus ioclados*	Poaceae	15.33% CL, 30.67% HC and 2.00% LG	Abideen et al., 2011
21	*Suaeda monoica*	Amaranthaceae	10.67% CL, 11.33% HC and 2.33% LG	Abideen et al., 2011
22	*Suaeda fruticosa*	Amaranthaceae	8.67% CL, 21.00% HC and 4.67% LG	Abideen et al., 2011
23	*Tamarix indica*	Tamaricaceae	12.17% CL, 24.67% HC and 3.33% LG	Abideen et al., 2011
24	*Typha domingensis*	Poaceae	26.33% CL, 38.67% HC and 4.67% LG	Abideen et al., 2011
25	*Urochondra setulosa*	Poaceae	25.33% CL, 25.00% HC and 6.33% LG	Abideen et al., 2011
26	*Miscanthus* spp.	Poaceae	40–60% CL, 20–40% HC and 10–30% LG	Brosse et al., 2012
27	*Achnatherum splendens*	Poaceae	16.7% LG	Xian-zhao et al., 2012

## Halophytes For Oil

Plant seed oil is a very good resource for the production of biodiesel. In fact, several plant species like canola, mustard, soybean etc. have already been explored for this purpose (Blackshaw et al., 2011). Many halophyte species (**Table 1A**) for example, *Suaeda aralocaspica, Salicornia bigelovii, Crithmum maritimum, Ricinus communis, Euphorbia tirucalli, Kosteletzkya virginica*, and *Descurainaia sophia* can store high concentrations of oils accounting for >20% of the total dry seed weight (Abideen et al., 2012, 2014). Studies show that the fatty acid methyl ester composition of oils extracted from halophytes is comparable to the other oil crops used for production of biodiesel (Abideen et al., 2012; Gul et al., 2013). The yield of oil varies in different species. *Ricinus communis* accumulates exceptionally high concentration of seed oils up to 40% of the total seed dry weight (Zhou et al., 2010; Abideen et al., 2014). Another halophyte species, *S. bigelovii* (pickle weed, sea asparagus or glasswort) produces about 2 ton seeds per hectare and can store oils up to 30% of the total dry weight (Glenn et al., 1999). *Euphorbia tirucalli*, a dicot plant, is estimated to have equal or even higher yields when grown on salt water than most of the oilseed crops irrigated using fresh water (Khaleghian et al., 2011). Perennial halophyte, *Kosteletzkya pentacarpos*, is also under investigation for biodiesel production from seeds and cellulosic ethanol from biomass (Moser et al., 2013). Its seeds contain about 20% oil and the disease susceptibility of the plant is very low. *Kosteletzkya virginica* is a perennial dicot halophyte with yields up to 1500 kg per hectare. Oil comprises of about 22% of the total dry weight of its seeds (Gallagher, 1985; Ruan et al., 2008). About 40 years ago, Melvin Calvin’s group had recommended *Euphorbia tirucalii* (a desert succulent) as a potential biofuel crop (Nielsen et al., 1977). It is currently being explored for its potential with saline sewage irrigation and has been shown to produce high amounts of secondary metabolites, which can be directly converted to biofuels (Eshel et al., 2010; Eschel et al., 2011; Hastilestari et al., 2013).

## Halophytes For Lignocellulosic Biomass

Lignocellulosic biomass is plant’s dry matter mainly comprising of cellulose, hemicellulose and lignin. In the recent past, several salt tolerant plant species have been identified with the potential to produce huge biomass on the salinity-affected lands (Qadir et al., 2008; Liu et al., 2012; Ventura et al., 2014). These include *Tamarix chinensis, Halopyrum mucronatum, Desmostachya bipinnata, Phragmites australis, Miscanthus sinensis, Phragmites karka, Typha domingensis* and *Spartina alterniflora* (Abideen et al., 2011; Liu et al., 2012). The perennials are usually preferred due to lesser cost of planting every year and longer canopy duration (Abideen et al., 2011).

*Tamarix* produces high biomass on non-arable soils irrigated with sewage and brackish water. Several species of *Tamarix* (*T. aphylla*, *T. jordanis* and *T. ramosissima*) have been reported to survive under harsh conditions of drought and salinity with biomass yields ranging from ∼20 to 52 t/ha/year (Eshel et al., 2010). Eschel et al. (2011) and Santi et al. (2014) tested several species of *Tamarix* for biomass production and cellulose content, and suggested *T. jordanis* as a promising candidate due to high cellulose content.

Lignin or its derivatives can inhibit or compromise the cellulase activity and therefore, pose major challenge for the conversion of biomass to ethanol. Therefore, plant species with less lignin in their cell walls are desirable and can reduce the cost involved in biofuel production. In a study conducted with the halophytes, found in the coastal regions of Pakistan, Abideen et al. (2011) reported perennial grasses, *Halopyrum mucronatum, Desmostachya bipinnate, Phragmites karka, Typha domingensis* and *Panicum turgidum* as potential species for bioethanol production. These species are estimated to contain <10% lignin content, 26–27% cellulose and 24–38% hemicellulose in their cell walls (**Table 1B**).

## Recent Developments And Opportunities

Clearly, biofuels offer a sustainable solution to address the environmental issues and would impact social as well as economic benefits. Especially, halophyte-based agriculture, where inputs are marginalized land and saline water from sea, has tremendous potential as biofuel feedstock. With their remarkable ability to tolerate salinity and limited energy costs associated with their production, halophytes have opened much barren land to agriculture. Adapting and improving the yield potential of these plants on the desert lands provide huge opportunities for the reclamation of the dry/saline lands like Sahara desert, Western Australia, Southern Africa, Thar desert and dry lands around the coastal regions. In addition, use of saline water for agriculture and vegetation in these dry lands will boost the ecophysiology, cool the environment, reduce heavy metal toxicity and help in establishing flora and fauna diversity (Lutts and Lefevre, 2015; Long et al., 2016). Moreover, halophytes are also very good experimental system to understand the molecular mechanism of salt tolerance and identify candidate genes for engineering salt tolerance in other conventional crops (Shabala, 2013). Recently, Song and Wang (2015) highlighted the potential of euhalophyte *Suaeda salsa* as a model system to explore the mechanism of adoption of dimorphic seeds to salinity environment.

An Initiative by Seawater Foundation for Greening Eritrea is a remarkable example of how a desert soil can be converted to a useful resource for agriculture (Ministry of Agriculture, 2002). Located on west coast of Red Sea, Seawater farms Eritrea were the world’s first commercial-scale integrated seawater farms. They had performed intercropping of *Salicornia* with mangroves (Ray and Anumakonda, 2011). Though the project was shut down because of repressive and xenophobic attitude of the government (Mission_2014, 2011); based upon the learning outcomes, Hodge developed another similar project at Mexico and founded Global Seawater Inc. for producing *Salicornia* based biofuels (Dickerson, 2008). Due to mismanagement of *Salicornia* production, Global Seawater Inc. could not provide enough biofuel to Mexican airlines for the trial in 2010. However, in the meantime, several private and government agencies realized the potential of halophyte-based biomass/food/feed production and made significant investments in this field (O’Keeffe, 2010). The National Aeronautics and Space Administration (NASA) of Unites States of America has constructed a GreenLab research facility for biomass optimization with viable options for alternative energy resources. The halophytic species of their primary focus are *Salicornia virginica* (grown widely), *S. bigelovii* (high lipid content), *S. euphoraea* (tall and large), *Rhizophora mangle* (Red mangrove), *Avicennia berminans* (Black mangrove) and sea grass (Bomani et al., 2011).

Several airlines have set up a Sustainable Aviation Fuel Users Group and are exploring halophyte-based biofuel production on the marginal lands through seawater agriculture^1^ (accessed August 21, 2016). Boeing, Etihad Airways and Masdar Institute of Science and Technology are working for the development of sustainable aviation biofuel. Their goal is to develop an Integrated Seawater Energy and Agriculture System (ISEAS) where biofuel feedstock will be cultivated with aquaculture and mangrove silviculture. After generating some breakthrough preliminary and exciting data, the consortium is developing about 2000 sq. km of the desert land in the western region of Abu Dhabi for halophyte farming where sea water will be used for irrigation (Cornwell, 2014). A pilot facility has already been built in this regard. New Nile Co. is also working on the implementation of an Integrated Seawater Agriculture System (ISAS^TM^) that combines the seawater and arid non-agricultural land to produce biofuels, animal fodder and other food products^2^ (accessed on August 21, 2016). *S. bigelovii* has been proposed as a halophytic species for integrated aqua-agriculture system as it can grow on aquaculture eﬄuents with lots of nutrients and water for irrigation. Further, it has been estimated to produce up to 900 liters of biodiesel per hectare land (Christiansen, 2008) The International Centre for Biosaline Agriculture (ICBA) also, based on their experiments on *S. bigelovii* in United Arab Emirates (UAE), highlighted its growing potential in Gulf cooperation council countries for biomass and seed production (Shahid and Rao, 2011). ICAB is actively working to identify wide range of salt tolerant crops, analyzing the yield of selected crops on the marginal lands and providing technical support to the farmers through National Agricultural Research Systems of the partner countries (Shahid et al., 2011).

However, in spite of all these successful results and ongoing efforts to screen and identify most suitable halophytic species, there are several challenges down the road for large-scale adoption of halophytes as feedstock/food crops. Firstly, identification of region-specific genotypes and adapting them in existing agricultural infrastructure without compromising their yield potential is required (Koyro et al., 2011; Ksouri et al., 2012; Buhmann and Papenbrock, 2013; de Vos et al., 2013; Katsching et al., 2013; Rozema and Schat, 2013; Shpigel et al., 2013). We need to launch appropriate breeding efforts as these plants have never been grown systematically in breeding trials for domestication (Zerai et al., 2010). For example, small size and uneven ripening of the seeds in *S. bigelovii* can lead to loss of up to 50% during harvesting therefore, improved mechanical harvesting technology is required. Many halophytic species are intolerant to high concentration of salts during germination and seedling establishment. Therefore, transplanting of seedlings or stem cuttings is practiced that needs optimum growth conditions, on-site fresh water resources and stock plant management.

Further, accumulation of inorganic salts in biomass that helps plants to adapt to saline environment can complicate both thermochemical and biochemical conversion for biofuel production. Efficient processing of halophyte-based biomass requires significant investments and efforts from the scientific community to increase saccharification efficiency, reduce ash content and development of other byproducts (Brown et al., 2014). Though modern genome editing tools or transgenic technologies can expedite the halophyte improvement for reduced accumulation of inorganic salts, we would need to ensure that tailoring halophytes for optimizing biofuel-related traits does not compromise on their ability to grow in salinity affected areas and, ward off pathogens and pests. Screening germplasm for the traits of interest would be a good approach to exploit already existing genetic diversity and select best genotypes.

However, the key experimental model systems namely, *Arabidopsis* and rice, for which extensive genetic and genomic resources have been developed, are glycopytes. With the advent of next generation sequencing technologies, whole genomes of candidate halophytes have been sequenced (Yang et al., 2013) while RNAseq followed by *de novo* assembly has been used for gene expression profiling in halophytes (Tsukagoshi et al., 2015; Wang et al., 2015; Jin et al., 2016). However, we need to generate concrete genetic and genomic resources for halophytic models to better understand the mechanism of salt tolerance and physiology of halophytes. Also, little information is available about the potential pathogens of halophytes when grown at large scales.

Overall, identification of region-specific halophytes, accessibility to seeds, development of halophyte nurseries, optimizing cultural practices (planting methods, fertilizer requirements, insect–pest management), establishment of processing plants and development of new landscape management system are immediate requirements to develop saline agriculture for bioenergy crops. Plant breeders and molecular biologists would have to join hands to expedite the process of incorporation of desirable changes in the plant makeup that are imperative for fuel, production of additional high value products and improve their yield potential in terms of oil content, seed yield and lignocellulosic biomass. Since, large-scale planting of halophytes has not been attempted, only time will unveil the practical challenges associated with halophyte farming and guide future research directions.

## Author Contributions

MS and RS conceptualised, prepared the framework and wrote the review. SW and VS collected the literature and prepared the table. AP helped in preparing the framework and finalising the article. All authors approved the article.

## Conflict of Interest Statement

The authors declare that the research was conducted in the absence of any commercial or financial relationships that could be construed as a potential conflict of interest.

## Footnotes

1 http://www.safug.org/

2 http://www.newnileco.com
